# Observations from the emergency management of dialysis patients evacuated from the US Virgin Islands to Puerto Rico following hurricane Irma

**DOI:** 10.1186/s12913-021-07194-6

**Published:** 2021-11-16

**Authors:** Guillermo J. Avilés Mendoza, Kristen P. Finne, Francisco Torre Leon, Lisandro Montalvo Burke, Jessica Cabrera-Marquez, Ana M. Mercado Casillas, Grasiela Malave, Christopher Brown, Jeffrey Kelman, Jeffrey B. Kopp

**Affiliations:** 1 Department of Health and Human Services, 200 Independence Avenue, Washington, DC 20201 USA; 2 Department of Health and Human Services, 200 C Street SW, Washington, DC 20515 USA; 3 Atlantis Health Care Group, CARR, 199 Avenue Las Cumbres, #140, Guaynabo Medical Mall, Bldg STE 107, Guaynabo, PR 00969 USA; 4 Medical Advisory Board, Fresenius Kidney Care, FMC San Juan Dialysis Center, 461 Calle Francia STE, A-101, Antillas Warehouse, San Juan, PR 00917 USA; 5 Office of Biosecurity, Puerto Rico Department of Health, 199 Ave Las Cumbres, #140, Guaynabo, Medical Mall Building Suite 107, Guaynabo, PR 00969 USA; 6 End Stage Renal Disease Network 3, Cranbury, NJ USA; 7 Center for Medicare, Centers for Medicare & Medicaid Services, U.S. Department of Health and Human Services, Washington, DC USA; 8 Department of Health and Human Services, Kidney Diseases Branch, National Institute Diabetes and Digestive and Kidney Diseases, National Institute of Health, Bethesda, MD 20892-1268 USA

**Keywords:** Disaster response, Special needs population, Access and functional needs, Disaster shelters, End-stage renal disease, dialysis, Hemodialysis, US public health service, Volunteers, End-stage renal disease network

## Abstract

Two category 5 hurricanes, Irma and Maria, arrived in the Caribbean in September 2017 in rapid succession. On September 6, Irma devastated the islands of St. Thomas and St. John, in the Virgin Islands of the United States (USVI). Most medical infrastructure was damaged, including hemodialysis facilities, paralyzing dialysis operations. After Irma’s landfall, Puerto Rico served as a safehaven for thousands of displaced and repatriated persons from the impacted islands. These included a cohort of 129 hemodialysis patients evacuated from St. Thomas, USVI to San Juan, Puerto Rico from September 9−11, 2017. The hemodialysis patients arrived first at hotels in San Juan and were then transferred to a Special Needs Shelter, run by the Commonwealth of Puerto Rico and located in the Puerto Rico Convention Center. With the imminent arrival of Hurricane Maria, most patients were evacuated on September 19 to a special needs shelter on the campus of the Florida International University, in Miami, Florida. While in San Juan, hemodialysis treatments were provided by local nephrologists working with local hemodialysis centers. Here, we describe the challenges and the emergency management actions taken to ensure continuity of care, including providing dialysis, general medical care, shelter, food and transportation for USVI dialysis patients during their stay in San Juan, Puerto Rico. We describe here the experiences of federal and host state/territorial officials in the special needs shelter, in the context of the state/territorial and federal response to disasters, in order to provide ideas about challenges, solutions, and approaches to coordinating care for dialysis patients evacuated from a disaster.

## Introduction

The 2017 Atlantic hurricane season was among the most active in history with nine hurricanes. Further, eight consecutive tropical storms reached hurricane intensity (without intervening storms of lesser intensity) in August and September, representing a first-time event in 124 years of record-keeping. Four hurricanes made landfall in the United States (U.S.): Harvey (Category 4, winds 130–156 mph), Irma (Category 5, winds ≥157mph), Jose (Category 3, winds 111–129 mph), and Maria (Category 4, winds ≥155mph).

Following the disasters, the affected populations included both general population individuals and at-risk populations with access and functional needs. Table 1 lists the major categories of the latter population, most of whom needed assistance with daily activities, either in the course of daily life or in the disrupted environment of a major disaster, and/or need on-going medical, nursing or other therapeutic interventions. Dialysis patients are a particularly vulnerable population, as many require continual access to healthcare services and medications due to their co-morbidities, a specialized diet and need for transportation to hemodialysis centers or a suitable environment and supplies for peritoneal dialysis. Elements of a plan to provide support for chronic hemodialysis patients are laid out in Table 2.

**Table 1:** At-risk population considerations in disaster settings

At-risk population	Medical needs	Shelter requirements
**Hemodialysis**	Hemodialysis 3 times per week	Transportation to/from dialysis facility For patients with grafts or fistulas, inform all staff to take blood pressure only in non-access arm and label bed or cot
**Peritoneal dialysis**	Private area, sterile field, dialysis solution, for chronic ambulatory peritoneal dialysis or a peritoneal dialysis cycler	Patients using a dialysis cycler may require hospitalization or special accommodations to provide sterile areas
**Renal transplant**	Immediate provision of immunosuppressive medication (within 24-48 h), to prevent allograft loss	
**Diabetes**	Insulin Glucose strips (urine, blood) Glucometer	Dedicated refrigerator for medications (not shared with food items)
**Limited or mobility impairment**	Wheel chair, walker, crutches, bed-bound	Access, wheelchair accessible Hospital beds and staff to turn patients who need particular kinds of help
**Families with children**		Segregated area, activities
**Cardiopulmonary disease**	Oxygen	
**Communicable disease e.g. active tuberculosis**	Isolation	Isolation is not feasible, and so must be hospitalized
**Cognitive impairment and mental illness**	Behavioral health and personal support services	Adequate staff: evacuee ratio Special attention to night shift
**Most evacuees**	Medications	Names, doses, and schedule may be unavailable

**Table 2:** Elements of a plan to care for hemodialysis patients in disaster settings

	Challenge	Approach
**Intake evaluation**	Prior history of illness, medications, surgeries, allergies, dialysis prescription will be needed	Some data will be available from the ESRD Network Develop intake form and make it readily available to the providers Copy form so that it is available immediately to providers and to administrative form
**Medications**	Evacuees may leave quickly with few or no medications Almost all ESRD patients are covered by Medicare and some are covered by Medicaid, but co-pays cannot usually be paid by evacuees	Engage local pharmacies to determine if they have a mechanism for donating medications Patients should be encouraged to contact their health insurance providers to determine if a “refill too soon waiver has been implemented to allow replacement. Determine if the HHS Emergency Prescription Assistance Program (EPAP) can be activated
**Dialysis**	Identifying facilities with capacity to absorb new patients	Help from ESRD Network
**Dialysis prescription**	May not be immediately available from ESRD network Dry weight may be unknown Blood chemistries are likely to be unavailable	Generate a standard dialysis prescription
**Transportation to/from dialysis facilities**	Three times per week May be four shifts	Contract for transport, including accessible buses and ambulances as a last resort
**Volunteers**	Confirm professional staff qualifications Supervise volunteers	
**Emergency response**	Personnel, equipment, transportation, road conditions	Paramedics and emergency medicine technicians 911 emergency response network
**Vascular access**	Catheters and grafts, thrombose Grafts and fistulas may narrow	Identify vascular access clinic
**Dietary needs**	Renal diet (low sodium, moderate potassium) Diabetic diet	
**Behavioral Health**	Anxiety, depression, grief Anger over loss of family, friends, companion animals, and loss of control, Disorientation in new environment, possibly new state	Phone calls to family are supported, if cell service is available Spontaneous patient group discussions Counseling by psychology and social work
**Communication**	orrC Coordinate dialysis schedules, response to acute medical and other needs	Teleconferences, Listserv
**Medical information and records**	Coordinate and share data on medical history, medications, allergies, acute conditions, schedules	Electronic medical record Spreadsheets Access from laptop computers

## Hurricane Irma and dialysis patients

Hurricane Irma was a category 5 hurricane that remained a hurricane from August 30 to Sept 17, 2017. The storm had winds that reached 185 miles per hour. Its sustained winds were the strongest since Hurricane Wilma in 2005. Winds, storm surge, and flooding caused 97 deaths, including 10 deaths in in the U.S. and damage is estimated at 50 billion dollars in U.S. [1]. The storm made landfall in Cuba and later Florida. Catastrophic damage occurred as the category 5 storm traveled and hit many of the Caribbean islands, French and British Virgin Islands and Saint Thomas and Saint John in the Virgin Islands of the United States (USVI). On St. Thomas, approximately 90% of homes and businesses were damaged and many lost roofs that could only be temporarily patched with tarps as they became available. For the most part, all of St. Thomas’ transportation, communication and public utility infrastructure (e.g.*,* power, water and transportation) were catastrophically damaged and led to the implementation of extended daily curfews for public safety [2]. Delays in supplies also resulted from St. Thomas’ small airport sustaining significant damage that led to temporary closure and limited operational capacity, ultimately delaying and restricting the volume of pre-staged federal and territorial disaster response assets for days. Individuals were also unable to self-evacuate as the damage prevented commercial flights from resuming service until September 28, 2017, and the ferry system was catastrophically damaged and inoperable.

Prior to the storm, St. Thomas had two hemodialysis units (Schneider Hospital Dialysis Facility and the Caribbean Kidney Center (CKC)) providing hemodialysis services for approximately 131 patients on the island (data from Centers for Medicare & Medicaid Services (CMS), End-Stage Renal Disease (ESRD) Network 3). Both facilities, as a protective health measure, provided early dialysis treatments in advance of Irma’s landfall, instructed their patients to implement an emergency renal diet [3] and to notify the facility of their status or preferably, if they could, visit their facility for treatment within 48 to 72 h after the storm.

Following the storm, almost none of the patients and staff were able to communicate by phone and many were stranded and unable to reach the dialysis units due to debris and damaged roads. Both dialysis units sustained damage during the storm. The Schneider Hospital and Dialysis Facility had significant damage and was only able to minimally support short dialysis treatments and print their patient dialysis medical plans before their potable water, technology and generator failed on Saturday, September 9, 2017. The disaster-impacted critical infrastructure, lack of access to acute health care services and transportation led to a rapidly deteriorating situation that necessitated life-saving evacuations, particularly as St. Croix’s healthcare facilities could not support the needs of both islands.

Given these circumstances, officials from the US Department of Health and Human Services (HHS), ESRD Network 3 and USVI and Puerto Rico governments rapidly developed and executed a plan to evacuate 129 of the 131 hemodialysis patients as two of the patients had alternate plans and did not require assistance. HHS, leveraging available federal helicopters and an air ambulance coordinated life-saving evacuations of these 129 dialysis patients from St. Thomas to San Juan, Puerto Rico during the period of September 9–11, 2017. HHS public health and medical emergency responders leveraged the HHS emPOWER Program [4], a joint Office of the Assistant Secretary for Preparedness and Response (ASPR) and Centers for Medicaid & Medicare Services program, to access administrative insurance claims data to obtain the physical addresses of the Medicare dialysis patients and obtained the remaining data via ESRD Network 3, using the CMS Consolidated Renal Operations in a Web Enabled Network (CROWNWeb) System [5] and information available from dialysis staff that were accessible by a satellite phone at the hospital. HHS National Disaster Medical System (NDMS) Disaster Medical Assistance Team (DMAT) members were embedded with Federal Emergency Management Agency (FEMA) Urban Search and Rescue Teams who used air and high-water vehicle assets to reach and rescue patients located across the island. Each patient was assessed by a DMAT member to determine their medical status, evacuated to the Schneider Hospital and then prioritized for evacuation off the island, along with the other acute medical evacuees, as the air assets could only carry a few patients at a time and fly safely during daylight hours. These significant limitations ultimately restricted family members, often serving as caregivers, from being able to accompany the dialysis patients during the evacuation. Patients were monitored for hyperkalemia and other potential complications and these findings were reported to the medical volunteers (nephrologist, nurses and paramedics) and the public health receiving team upon arrival at the San Juan Airport. The team rapidly reassessed each patient and triaged them to a hospital if they had an acute medical need. Those only requiring dialysis were transported to one of the Atlantis Dialysis or Fresenius Medical Care facilities that had volunteered to accept and coordinate treatment for the duration of the patients’ evacuation. Those determined to be stable and who had completed their dialysis treatment were then taken to one of the hotels that the Puerto Rico Department of Public Health (PRDOH) had arranged as temporary housing while they identified a shelter location and coordinated support resources.

The PRDOH issued a call for action for medical volunteers to temporarily support the patients in the hotels and to later staff the special needs shelter that they established in San Juan. HHS DMAT members and United States Public Health Service (USPHS) Officers formed a medical assessment team and went door to door in the hotel to assess each patient, compile medical histories and obtain medication lists, using a two-page paper form. To ensure continuity of care, patients were assigned to either the Atlantis or Fresenius dialysis facility for all ongoing dialysis treatments and care coordination.

## Coordination of the response: USVI Dialysis patient response taskforce in Puerto Rico

On September 11, HHS and PRDOH rapidly established the USVI Dialysis Patient Response Taskforce. Leadership was shared by two federal officials and a lead official from the PRDOH. Other task force members included medical personnel from local hemodialysis units; local nephrologists who took charge of overseeing hemodialysis, medical care and coordination of these patients; ESRD Network 3 staff; HHS personnel and others.

A sample battle rhythm of the task force is displayed in Table 3**,** and started with a daily synchronization briefing at 0700, followed by twice daily task force meetings at 0800 and 1700 h. (The military term “battle rhythm” describes the daily cycle of command and staff activities that synchronizes daily and future activities.) At the end of each day, the Task Force leads provided a daily situational report to the Federal Health Coordinating Official of the Emergency Support Function (ESF)-8 response and identified needs were taken for action and completed, as possible, by the next shift. For example, HHS staff worked with FEMA to execute a Personal Assistance Services Contract to hire local personal aides, who would support the many patients who required assistance with activities of daily living. They also coordinated program and policy waiver reviews and identified alternate options and access to critical health services, including prescription medications, from numerous federal agencies. The CMS ESRD Program and ESRD Network 3 leadership and staff also worked to coordinate stateside access to dialysis care for patients that had the ability and resources to self-evacuate from San Juan to alternate locations where they had family and friends who could assist them.

**Table 3:** Daily schedule for dialysis task force caring for US Virgin Islands patients

Time	Activity
0630	Situational report from Puerto Rico Department of Health
0700	USPHS Incident Response Command Team teleconference, led by the Federal Health Coordinating Official – Federal dialysis task force provides situation report
0730	Federal dialysis task force team meeting
0800	Federal dialysis task force teleconference with national dialysis providers and local nephrologists
0900	Morning rounds at FMS, with PHS officers, nursing staff and volunteers
1100	HHS Public Health and Medical National teleconference
1300	Situational report with Puerto Rico Department of Health on 1200 dialysis shift
1630	Situational report with Puerto Rico Department of Health on 1600 dialysis shift
1700	Federal dialysis task force teleconference with national dialysis providers and local nephrologists
1900	Evening rounds at FMS, with PHS officers, nursing staff and volunteers
2000	Federal dialysis task force sends situational report to Federal Health Coordinating Official and Puerto Rico Department of Health team lead and actions were identified and coordinated for the next shift

The daily schedule of the Federal dialysis task force included team meetings and clinical rounds, and teleconferences with local and federal response teams and local nephrologists, dialysis units, and national dialysis providers (Atlantis and Fresenius). The calls enabled coordination of transportation to and from dialysis units, with up to three shifts each day**.** USPHS; special needs shelter

**Fig. 1:**
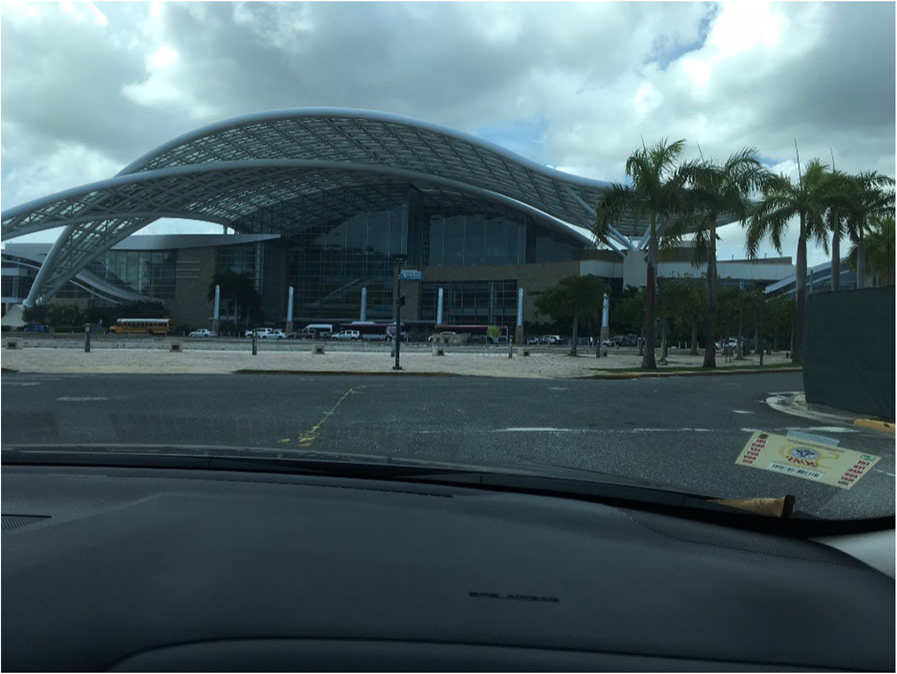
The Puerto Rico Convention Center Dr. Pedro Rosselló González. The Puerto Rico Convention Center opened in 2005 and has an exhibit hall with an area of 14,600 m2. The center was used to house the dialysis patients evacuated following hurricane Irma. This picture was taken as the patients were inside and the advance clouds of hurricane Maria appeared. Following the arrival of hurricane Maria, the convention center has functioned as a warehouse and special needs center to support relief efforts

On Sept 15, the remaining dialysis patients and a few caregivers, who were able to travel with the last group of evacuated patients, were moved to a Commonwealth of Puerto Rico Shelter that was established at the Puerto Rico Convention Center (Fig. 1). This facility housed both general population individuals and medical needs patients, most of whom were the dialysis patients. General population shelters are usually run by a state, territorial or a non-government organization. Individuals that require medical assistance and services but do not meet hospitalization requirements may be placed within a selected area of the general population shelter or in a dedicated special needs shelter. Special needs shelters are usually established, managed and staffed by the state or territorial department of health and may submit an official request to the federal government to provide DMAT and USPHS officer medical staff augmentation. In recent years, it has become apparent that dialysis patients comprise a large portion of medical needs patients who need substantial assistance following disaster-induced displacement from their home, including assistance with coordination of care, personal assistive and transportation services, as described [6]. In disasters that result in significant geographic evacuation, such as Hurricanes Katrina and Rita, state and territorial governments can enter into host state agreements, enabling FEMA to reimburse mutually agreed upon services that are provided to evacuees temporarily residing in the host state or territory. Widespread catastrophic damage to USVI’s critical infrastructure significantly impeded communication and coordination, including support agreements amongst state and territorial partners in the initial months of the response.

A portion of the ground floor of the convention center was laid out as a grid containing 120 cots. Two USPHS officers served as liaisons to shelter management to assist with coordination and integration of officially requested federal resources in the shelter. Personnel from the PRDOH provided 24-h coverage and consisted of two physicians and two registered nurses, each working 12-h shifts. Staffing was augmented by Puerto Rico Medical Reserve Corps (MRC) volunteers, as well as student nurses with their supervisor and general volunteers. Evening coverage was supplied by a six-person team composed of contract paramedics and DMAT doctor and nursing staffs. HHS requested a FEMA contracted team of paramedics and emergency medical services personnel to augment shelter medical support for the patients. This team arrived on the third night. Meals following the American Diabetes Association dietary guidelines for ESRD patients were provided to the patients by the convention center staff.

On Sept 19, with the imminent arrival of Hurricane Maria, 90 patients were evacuated within hours, on short notice, by a chartered jet to an alternate special needs shelter. This shelter was located in a sports arena on the campus of the Florida International University in Miami and was staffed by the USPHS Rapid Deployment Force-2. The initial characteristics of these patients were as expected for a hemodialysis population, with many being older adults and having multiple co-morbidities, including diabetes. This population also had additional significant access and functional needs that included but were not limited to individuals with mobility needs (i.e. amputees, double amputees, bed-bound, wheelchair, walker, etc.), impaired vision or blindness, cognitive deficits, behavioral health needs, and oxygen dependency. The patients remained in this shelter for an additional week until temporary housing in hotels and dialysis facilities that could accommodate the need were identified in Atlanta, Georgia. Patients were evacuated by flight to the hotels and federal agencies coordinated the logistical support requirements (e.g. access to dialysis, transportation, disaster case management, etc.) through federal assistance programs until the dialysis facilities could be replaced and or repaired on St. Thomas and St. Croix a year later.

## Analysis of the response

A number of issues proved challenging and the ways in which they were addressed may provide lessons for future disasters where responders need to manage a large number of displaced dialysis patients, together with caregivers.

### Medical information and records

Complete medical histories and electronic medical records were not readily available for the providers in the shelters. The medical history information collected by DMAT members was entered into NDMS electronic medical records and similarly provided to the dialysis providers to input it into their own systems. Providers in the shelters were not able to access this information and separately managed patients using a binder that contained all their patient medical forms. New events were recorded on additional pages.

The lack of complete records required an initial in-person evaluation by local nephrologists prior to an individualized dialysis plan and order being generated at the unit. This was challenging as some patients had not received full dialysis treatments for several days, some had uncontrolled blood pressure values that needed to be managed prior to connection and some had evidence of fluid overload.

This experience further highlights the critical need for nationwide health information portability and interoperability via health information exchanges (HIEs), particularly in disasters, as well as the ability to rapidly deploy secure disaster response health information technology platforms. For example, the Patient Unified Look-Up System for Emergencies (PULSE) [7], is a public-private data collaborative, that can be adopted and deployed at the state or local level to rapidly authenticate and enable credentialed disaster healthcare volunteers to securely access, query and view patient records, such as dialysis plans, from all connected HIEs, providers and organizations. Successful deployments of this platform in prior disasters has expedited and better ensured continuity of care for disaster impacted individuals.

### Case management and communications

A team of more than ten local nephrologists came together to assume responsibility for the provision of dialysis, additional care coordination and admission to local hospitals when medically necessary. It was vital that there be regular and effective communication among task force members including the shelter staff and hemodialysis unit staff. A core group consisting of two local nephrologists, ESRD Network 3 staff and others coordinated dialysis treatments and medical care of these patients. This was facilitated in several ways. As previously mentioned, teleconferences were held twice a day, at 0800 and 1700, and a listserv was created to disseminate information, including patients’ specific needs and concerns. As San Juan was not substantially affected by Hurricane Irma, the local telecommunications system was intact. The staff relied on laptop computers to maintain records and communicate by secure email or secure fax. When secure email was not feasible, documents were hand-carried from the dialysis patients to the PRDOH Emergency Operations Center, a single physical location where emergency response personnel coordinated the emergency response operation.

### Referral networks

Due to storm impacts on USVI, all patients were largely cut off from their healthcare providers and some patients experienced complications, such as compromised dialysis access (intravascular catheter or arteriovenous fistula or graft) that necessitated immediate medical referrals and treatment. In order to address these problems, the dialysis facility medical directors and case managers, in San Juan, established referral networks for specialty care and coordinating all other outpatient health provider and inpatient healthcare services for each of their newly acquired patients. These individuals also rounded on patients temporarily admitted to local hospitals and tracked them to ensure they resumed treatment and were monitored upon discharge from the hospital. This additional level of support was critical to stabilizing and mitigating likely disaster-induced adverse health outcomes in these patients and ensuring their safe return to their shelter location. A significant challenge that was not satisfactorily resolved was how reimbursement would be made to local hospitals and vascular access centers and copays were challenging for the patients. While most of the patients had Medicare coverage, many were also enrolled in the USVI Medicaid Program or other secondary insurance programs that had separate administrative processes that were required to obtain reimbursement for services. Administrative enrollment and initiation of these processes was delayed due to USVI systems being impacted and offline for extended periods of time. Public and private insurance and other service programs should review their continuity of operations plans to ensure they have redundant policies, processes and back-up systems that can address disaster scenarios that may include large scale out-of-state or territory evacuations and expedited enrollment of out-of- network providers that are critical to ensuring continuity of care for health and human service dependent populations [8].

### Transportation

A major administrative task was scheduling and adjusting transportation, on a daily basis, for two separate dialysis units that each had multiple dialysis treatment shifts each day, and two separate cycles per week (Monday-Wednesday-Friday and Tuesday-Thursday-Saturday). The Puerto Rico Agency for Public Transportation transported the patients by bus for one of three dialysis shifts: 06:00, 12:00 and 16:00 on Monday-Wednesday-Friday or Tuesday-Thursday-Saturday at an Atlantis or Fresenius Medical Care dialysis unit. Puerto Rico MRC staff coordinated appointments with the facilities and were responsible for ensuring that patients were fed and on the buses at the appointed times. To simplify transportation, patients were dialyzed on a limited number of shifts, and local dialysis patients graciously accepted changes in their dialysis schedules.

### Medications

Many of the patients and some caregivers evacuated with a limited supply of or no medications at all. This rapidly became problematic, as many depended on them to control their diabetes, hypertension, heart disease and other conditions. The CMS issued a provider bulletin that outlined flexibilities under declared disasters for accessing prescriptions and also expectations that “refill-too-soon edits” were expected to be lifted. Local pharmacies in San Juan had difficulty verifying patient eligibility for Medicaid, as the USVI Medicaid system sustained damage and was offline. While most patients were Medicare and Medicaid beneficiaries, many were unable to cover their copays, which created additional challenges. HHS, in partnership with the United States Department of Veterans Affairs (VA), created a mechanism to provide critical prescription medications for the dialysis patients. Local pharmacy chains also donated prescriptions to help as federal law prohibited co-pay waivers. Access to essential medications following disasters continues to be a significant challenge. Additional planning and consideration is needed to address challenges for future disasters, for example, government agencies and private sector partners could pre and post-disaster deploy medication caches and mobile pharmacy units to the disaster impacted areas to better ensure access to essential medications. Lastly, state and territorial government officials can also officially request activation of the HHS Emergency Prescription Assistance Program (EPAP) [9] that can provide access to free prescription medications, certain medical supplies, vaccinations and some forms of medical equipment to individuals that are eligible and resided in a federally identified disaster area.

### Logistics

Special needs shelters are often provided with a defined cache of supplies. Lacking a cache, needs were assessed and requests were submitted to local, state/territorial and federal agencies. Refrigerators were needed, one for food and one for insulin. Essential medical and cleaning supplies were needed, including exam gloves, medical-pathologic waste boxes, and needle disposal boxes for those using insulin. Building on these lessons learned, government and non-governmental agencies should, where possible, pre-deploy caches and expand current cache inventories to include medications and supplies commonly used by dialysis patients, particularly those that are diabetic, and ensure there is adequate cold storage and resources to store and dispose of medical waste.

### Medical conditions

Post-evacuation acute medical conditions included a hypoglycemic episode, managed with a glucose infusion, and an acute cellulitis that resulted in the patient being transported to a local hospital. Patients with pre-existing acute medical conditions who were determined to not be medically stable for patient movement from Puerto Rico to Florida were moved to local hospitals, ahead of Hurricane Maria’s landfall.

### Social support

Recognizing the psychosocial impacts of the dialysis patient evacuation, both dialysis facilities had several patients who volunteered to serve as greeters and visit with the dialysis evacuees during their treatments. Staff also sought, when possible, to provide status updates on the patients to their family members and or relay it to family members via their former dialysis unit nursing staff that were able to be reached by cell or satellite phones. The patients found these actions comforting and helped with their transition to their new facility for the duration of their stay. One of the many lessons learned is that a caregiver should accompany a dialysis patient and other chronically ill patients, whenever possible, throughout the period of displacement. This person can help by providing a medical history and in assisting the patient with activities of daily living.

### Language

The patients generally spoke only English, while many of the nurses and nursing students only spoke Spanish. Interpreters were found, particularly among volunteers arriving at the convention center. While options were quickly identified, the experience highlighted the importance of fully integrating cultural and linguistic considerations into all emergency preparedness planning, response, and recovery activities [10, 11].

### Discharge

During the time that patients stayed at the hotels and at the Puerto Rico Convention Center, there was attrition, as dialysis patients left to stay with family members in other locations. To assist these patients, ESRD Network 3 located dialysis facilities and coordinated patient medical information and other support to expedite care coordination and dialysis treatment upon their arrival. HHS regional staff and USPHS Service Access Teams, who were collocated in shelters, provided discharge planning assistance and connected patients to home and community-based services that included accessible housing, home health care, and personal assistive and other services.

## Discussion

The dialysis response to hurricanes that affected the U.S. mainland, including Hurricanes Katrina (2005), Gustav and Ike (2008) [12] and Super-Storm Sandy (2012) [13–16], have been the subject of analysis and commentary. Hurricane Katrina, which reawakened America to the threats posed by a major hurricane, prompted a particularly rich literature, addressing the effects of the storm on dialysis patients [17, 18], the need for planning and communication, the experience in general population and special needs shelters [19, 20], the role of stress on patients and on staff [21, 22], and lessons learned for the nephrology community [23–25].

During their seven-day stay in Puerto Rico, no dialysis patients died and pre-existing and post-evacuation identified medical problems were rapidly addressed and closely monitored throughout the patients stay in Puerto Rico. Regular hemodialysis was provided, using the parameters that had been established for each patient. Medications were provided, for the most part, although issues of reimbursement remained a challenge. That said, there were major shortfalls in the initial hours of care, particularly the ability to quickly provide replacement medications and the ability to generate medical records that could be accessed and modified by shelter staff and local nephrologists and dialysis facility staff. A secure electronic health record system or exchange and web-based access to prescription data, which could be accessed by multiple laptop computers provided to credentialed shelter staff and healthcare volunteers, would have eased coordination and facilitated communication among shelter staff, local nephrology staff, dialysis facility staff, local pharmacy staff, ESRD Network staff and federal officials and health care providers.

Ideally population shelters are set up in areas that are well outside of the predicted disaster zone. It is important to note that there is tension between the natural desires of individuals to stay in a familiar area with family and friends nearby as opposed to emergency response officials that seek to move individuals to more distant locations. These challenges are further compounded by an individual’s ability to self-evacuate due to a lack of resources and or transportation prior to and following a disaster. In the case of Hurricane Irma, widespread damage and the catastrophic loss of healthcare infrastructure necessitated the evacuation of dialysis patients off the island to Puerto Rico and later Miami, Florida, to ensure their continuity of care and access to dialysis services.

Dialysis unit staff can prepare for a disaster in several ways, as reviewed recently Bonilla-Felix, Blood Purification, 2019). A comprehensive disaster plan must involve staff, patients, families and cargivers. It should envision scenarios in which dialysis can continue at patients’ usual hemodialyis centers and scenarios in which patients must receive dialysis elsewhere, including relocating to centers outside the disaster zone. Peritoneal dialysis patients may continue to perform dialysis at home or in a special needs shelter, if they have adequate supplies Patients should prepare detailed plans that address transportation, medications, food supply and accessing emergency care. Patients may require assistance with transportation to the hemodialysis facility. Plans for hemodialysis unit staffing should address likely staff availability and options to bring on additional staff, including accessing state and local public health resources. Dialysis unit water quality may need to be confirmed. Adequate dialysis supplies should be on hand. Hospitalization may be required but this may occur at a time when hospital resources are stretched thin and emergency care is prioritized [26, 27].

Advances in technology and innovation are also critical to better ensuring the capacity and capability to deliver dialysis in disaster conditions. In 2018, ASPR launched the ASPR Next Program [28] to revolutionize disaster care with next generation technology. This program has since developed next generation portable hemodialysis machines that can deliver dialysis in disaster conditions. This investment, most recently, was used to provide dialysis surge capacity in hospitals during COVID-19 [29–31].

Each disaster provides new challenges and new opportunities to improve our emergency response to them. The historical phrase “never let a crisis go to waste” reminded us that we too must learn from of our collective experiences from the 2017 hurricanes by addressing lessons learned and adopting innovative approaches that can improve dialysis patient care and coordination in future disasters.

## Conclusions

With the advent of global warming, hurricanes are becoming more frequent and more severe, with more rainfall, stronger winds, and larger storm surges. The Saffir-Simpson hurricane wind was developed in 1971 and can applied retrospectively. Thirteen Atlantic hurricanes have reached category 5 intensity since 1924, with the following hurricane numbers over the past 7 decades: 1950s, 1; 1960s, 1; 1970s. 3; 1980, 1, 1990, 1; 2000s, 2; 2010s, 4. As a nation, we must be prepared for more severe weather events and ensure that we have adequate resources of personnel, material, and facilities to accommodate evacuees and attend to their needs. Systems are in place to deploy and coordinate resources at the local, state, territorial and federal levels, but a philosophy of continuous quality improvement in emergency response is required to ensure the best care for all individuals, including at-risk patients such those receiving dialysis.

## Data Availability

Not Applicable.

## Abbreviations

CKC: Caribbean Kidney Center
CROWNWeb: Consolidated Renal Operations in a Web Enabled Network
DMAT: Disaster Medical Assistance Team
ESF: Emergency Support Function
ESRD: End-Stage Renal Disease
FEMA: Federal Emergency Management Agency
HIEs: Health Information Exchanges
KCER: Kidney Emergency Response Coalition
MRC: Medical Reserve Corps
NDMS: National Disaster Medical System
ASPR: Office of the Assistant Secretary for Preparedness and Response
PULSE: Patient Unified Lookup System for Emergencies
PR: Puerto Rico
PRDOH: Puerto Rico Department of Health
HHS: United States Department of Health and Human Services
USPHS: United States Public Health Service
USVI: United States Virgin Islands
VA: United States Department of Veterans Affairs

## References

[1] U.S. Billion-Dollar Weathers & Climate Disasters 1980-2020. National Oceanic and Atmospheric Administration. https://www.ncdc.noaa.gov/billions/events.pdf.

[2] Dalton M, Althaus D. Irma death toll rises to 38 in Caribbean. The Wall Street Journal September. 2017;11.

[3] Kidney Community Emergency Response (KCER) Coalition. 3-day Emergency Kidney Diet. https://www.kcercoalition.com/en/resources/patient-resources/during-an-emergency/3-day-emergency-kidney-diet2/. Accessed 31 Aug 2018.

[4] U.S. Department of Health and Human Services. HHS emPOWER Program. https://empowerprogram.hhs.gov/. Accessed 17 Mar 2019.

[5] Centers for Medicare & Medicaid Services Consolidated Renal Operations in a Web Enabled Network (CROWNWeb). http://mycrownweb.org/help/about-crownweb/. Accessed 17 Mar 2019.

[6] U.S. Department of Health and Human Services. Personal Assistance Services to General Population Shelters Factsheet. http://www.phe.gov/Preparedness/planning/abc/Pages/pas.aspx. Accessed 31 Aug 2018.

[7] Patient Unified Lookup System for Emergencies (PULSE). https://sequoiaproject.org/pulse/. Accessed 17 Mar 2019.

[8] Merchant RM, Finne K, Lardy B, Veselovskiy G, Korba C, Margolis GS, Lurie N (2015). State of emergency preparedness for US health insurance plans. Am J Manag Care.

[9] U.S. Department of Health and Human Services Emergency Prescription Assistance Program (EPAP). https://www.phe.gov/Preparedness/planning/abc/Pages/pas.aspx. Accessed 17 Mar 2019.

[10] U.S. Department of Health and Human Services Cultural and Linguistic Competency in Disaster Preparedness and Response Fact Sheet. https://www.phe.gov/Preparedness/planning/abc/Pages/linguistic-facts.aspx. Accessed 17 Mar 2019.

[11] U.S. Department of Health and Human Services Ensuring Language Access and Effective Communication During Response and Recovery: A Checklist for Emergency Responders. https://www.hhs.gov/sites/default/files/lang-access-and-effective-comm-checklist-for-emergency-responders.pdf. Accessed 17 Mar 2019.

[12] Kleinpeter MA (2009). Disaster preparedness of dialysis patients for hurricanes Gustav and Ike 2008. Adv Perit Dial.

[13] Kelman J, Finne K, Bogdanov A, Worrall C, Margolis G, Rising K, MaCurdy TE, Lurie N (2015). Dialysis care and death following hurricane Sandy. Am J Kidney Dis.

[14] Lurie N, Finne K, Worrall C, Jauregui M, Thaweethai T, Margolis G, Kelman J (2015). Early Dialysis and adverse outcomes after hurricane Sandy. Am J Kidney Dis.

[15] Lempert KD, Kopp JB (2013). Hurricane Sandy as a kidney failure disaster. Am J Kidney Dis.

[16] Teperman S (2013). Hurricane Sandy and the greater New York health care system. J Trauma Acute Care Surg.

[17] Johnson DW, Hayes B, Gray NA, Hawley C, Hole J, Mantha M (2013). Renal services disaster planning: lessons learnt from the 2011 Queensland floods and North Queensland cyclone experiences. Nephrology (Carlton).

[18] Anderson AH, Cohen AJ, Kutner NG, Kopp JB, Kimmel PL, Muntner P (2009). Missed dialysis sessions and hospitalization in hemodialysis patients after hurricane Katrina. Kidney Int.

[19] Kutner NG, Muntner P, Huang Y, Zhang R, Cohen AJ, Anderson AH, Eggers PW (2009). Effect of hurricane Katrina on the mortality of dialysis patients. Kidney Int.

[20] Saunders JM (2007). Vulnerable populations in an American red Cross shelter after hurricane Katrina. Perspect Psychiatr Care.

[21] Brodie M, Weltzien E, Altman D, Blendon RJ, Benson JM (2006). Experiences of hurricane Katrina evacuees in Houston shelters: implications for future planning. Am J Public Health.

[22] Curtis A, Mills JW, Leitner M (2007). Katrina and vulnerability: the geography of stress. J Health Care Poor Underserved.

[23] Hyre AD, Cohen AJ, Kutner N, Alper AB, Dreisbach AW, Kimmel PL, Muntner P (2008). Psychosocial status of hemodialysis patients one year after hurricane Katrina. Am J Med Sci.

[24] Kenney RJ (2007). Emergency preparedness concepts for dialysis facilities: reawakened after hurricane Katrina. Clin J Am Soc Nephrol.

[25] Kopp JB, Ball LK, Cohen A, Kenney RJ, Lempert KD, Miller PE, Muntner P, Qureshi N, Yelton SA (2007). Kidney patient care in disasters: lessons from the hurricanes and earthquake of 2005. Clin J Am Soc Nephrol.

[26] Dialysis Centers. Topic Collection. Technical Resources. Office of the Assistant Secretary for Preparedness and Response (ASPR) Technical Resources Assistance Center Information Exchange (TRACIE) Website. https://asprtracie.hhs.gov/technical-resources/50/dialysis-centers/47. Accessed 24 June 2021.

[27] North Help Coalition. A Guide to Emergency Preparedness for Dialysis Patients. https://asprtracie.hhs.gov/technical-resources/resource/5417/a-guide-to-emncy-preparedness-for-dialysis-patients. Accessed 24 June 2021.

[28] ASPR Next. https://www.phe.gov/ASPRNext/Pages/default.aspx.

[29] HHS sends dialysis machines to hospitals with high cases of COVID-19; Fresenius ships equipment from Europe. Healio Nephrology News & Issues. https://www.healio.com/news/nephrology/20200513/hhs-sends-dialysis-machines-to-hospitals-with-high-cases-of-covid19-fresenius-ships-equipment. Accessed 21 June 2021.

[30] Tablo Hemodialysis Systems Deployed to Guam Hospitals by U.S. Department of Health and Human Services for Emergency Response. Yahoo News. https://www.yahoo.com/entertainment/tablo-hemodialysis-systems-deployed-guam-130000700.html. Accessed 24 June 2021.

[31] Kopp JB, Ball LK, Cohen A, Kenney RJ, Lempert KD, Miller PE, Muntner P, Qureshi N, Yelton SA (2007). Kidney patient care in disasters: emergency planning for patients and dialysis facilities. Clin J Am Soc Nephrol.

